# Secondary choroidal neovascularization due to choroidal osteoma after 9 years follow-up

**DOI:** 10.1186/s12886-021-02004-3

**Published:** 2021-05-31

**Authors:** Yun Zhang, Jia Fang, Shixin Zhao, Xiangjun She, Jun Wang, Lijun Shen

**Affiliations:** 1 Affiliated Eye Hospital of Wenzhou Medical University, Hangzhou, Zhejiang China; 2 Yongkang First People’s Hospital of Hangzhou Medical College, Jinhua, Zhejiang China

**Keywords:** Choroidal osteoma, Secondary choroidal neovascularization, 9 years follow-up

## Abstract

**Background:**

Choroidal osteoma is a benign intraocular tumor that can increase risk of developing choroidal neovascularization. The visual prognosis is influenced by the tumor location, decalcification status, overlying RPE atrophy, presence of choroidal neovascularization, persistence of subretinal fluid and occurrence of subretinal hemorrhages.

**Case presentation:**

The authors present a 40-year-old woman diagnosed with choroidal osteoma of the right eye. Her best corrected visual acuity was 12/20 but decreased to 5/20 due to secondary choroidal neovascularization after 8 years follow up. Fundus examination revealed an enlarged choroidal osteoma in most margins at posterior pole with schistose hemorrhage beside macula. Optical coherence tomography angiography revealed unique features in the vascular changes of choroidal neovascularization in choroidal osteoma in the outer retinal layer and choroid capillary layers, and subretinal neovascularization. Indocyanine green fluorescence angiography showed there was hypo-fluorescence at the peripapillary with faint hyper-fluorescence at the macular, corresponding to the location on the fundus photograph. The patient received 3 injections of intravitreal ranibizumab. After 1 year follow up, her visual acuity of the right eye was 18/20 and the CNV had regressed.

**Conclusions:**

We present the findings and treatment of a case of choroidal osteoma with secondary choroidal neovascularization. Optical coherence tomography angiography combined with FFA and ICGA is used to analysis the characteristics of secondary choroidal neovascularization. Optical coherence tomography angiography can reveal some unique characteristics in the vascular changes compared to fundus fluorescein angiography.

## Background

Choroidal osteoma(CO) is a rare, benign, ossifying tumor within the choroid of unknown etiology [1]. CO is always found in young healthy females about 20 or 30 years old, with no history of systemic or ocular disease. 80% of cases is unilateral clinically and tends to occur choroidal neovascularization(CNV) in up to one-third of cases [2]. The initial descriptions of choroidal osteoma was by Gass in 1978 [3], as a slightly elevated, juxtapapillary, yellowish orange, choroidal lesion with well-defined margins. Fundus fluorescein angiography (FFA), indocyanine green fluorescence angiography (ICGA) and optical coherence tomography angiography (OCTA) as ancillary diagnostic tests may help demonstrate choroidal osteoma and detect RPE atrophy, CNV formation and the characteristic spider vessels, which may be amenable to treatment by directing for complications arising from CNV and subretinal fluid.

In this case report we using multimodal imaging to describe the change of this disease after 9 years follow-up, especially the secondary choroidal neovascularization.

## Case presentation

**Fig. 1:**
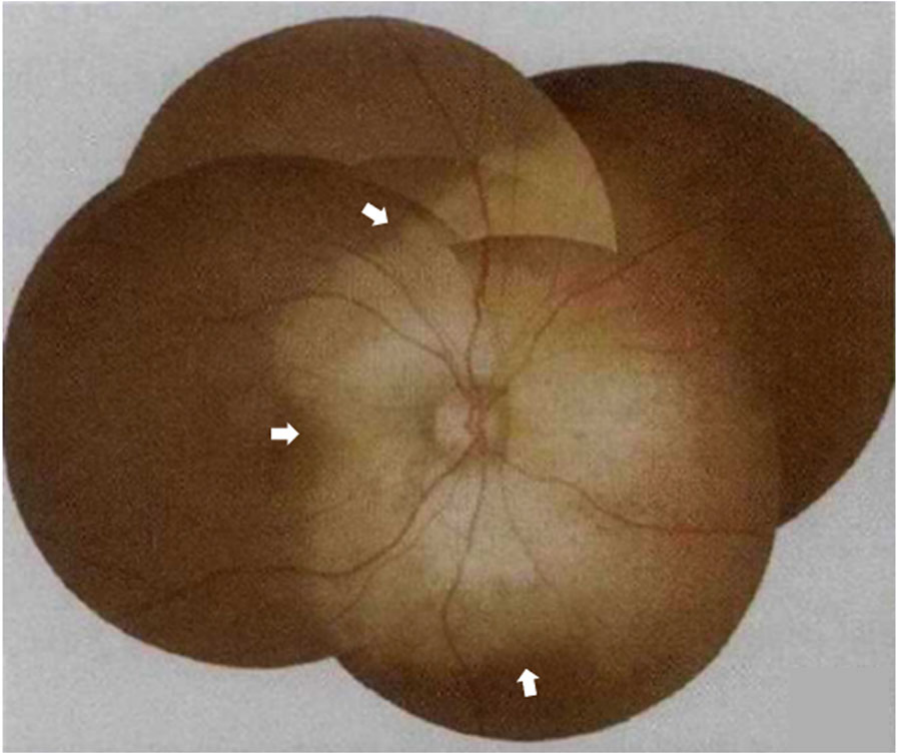
Fundus photograph showed a yellowish white, peripapillary and sharply demarcated choroidal lesion involving the macula (white arrows)

**Fig. 2:**
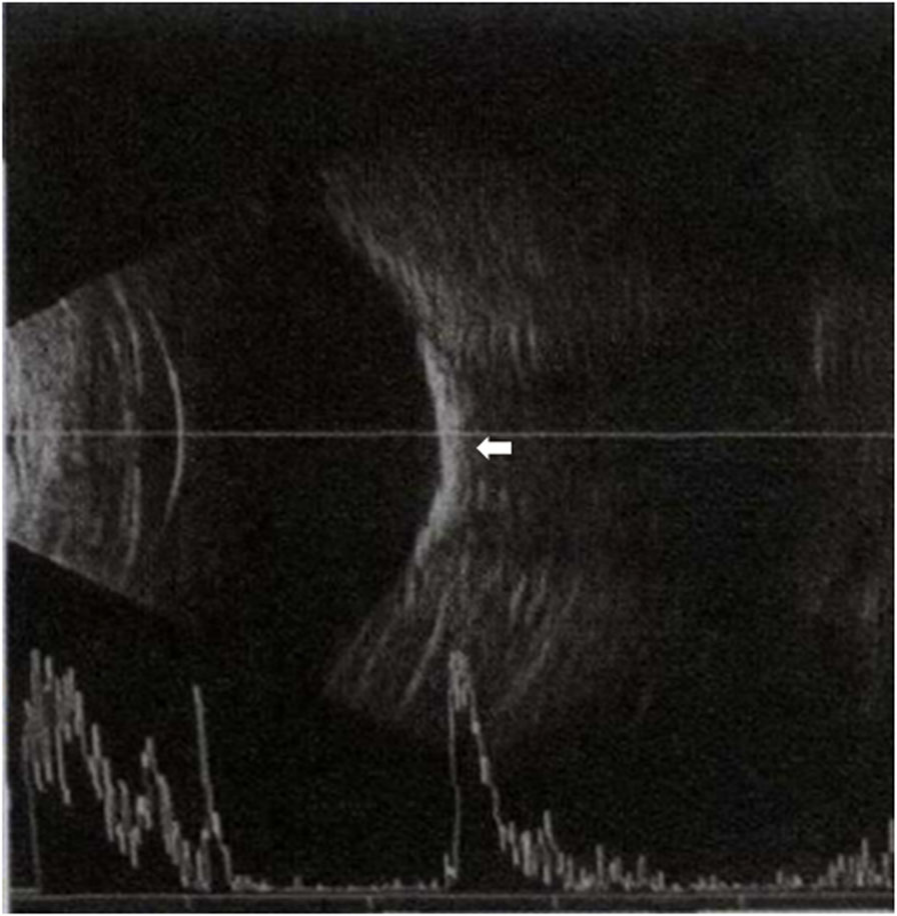
B-scan demonstrated focal subretinal calcification next to the optic disc (white arrow)

**Fig. 3:**
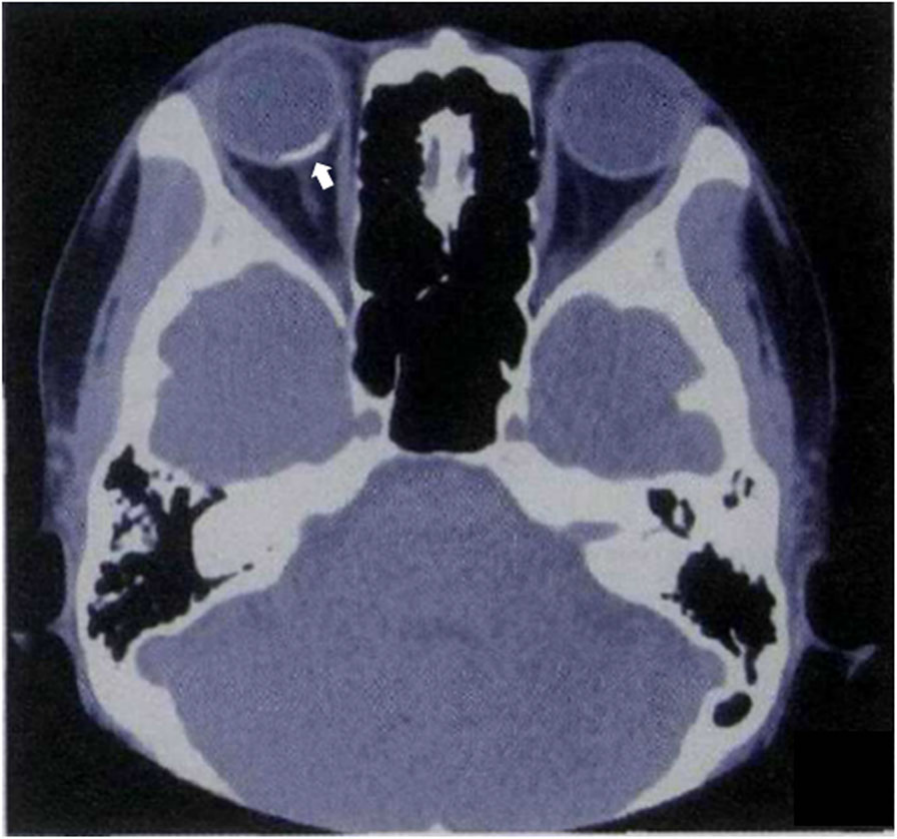
CT demonstrated a hyperdense choroidal plaque with the same density as bone typically (white arrow)

**Fig. 4:**
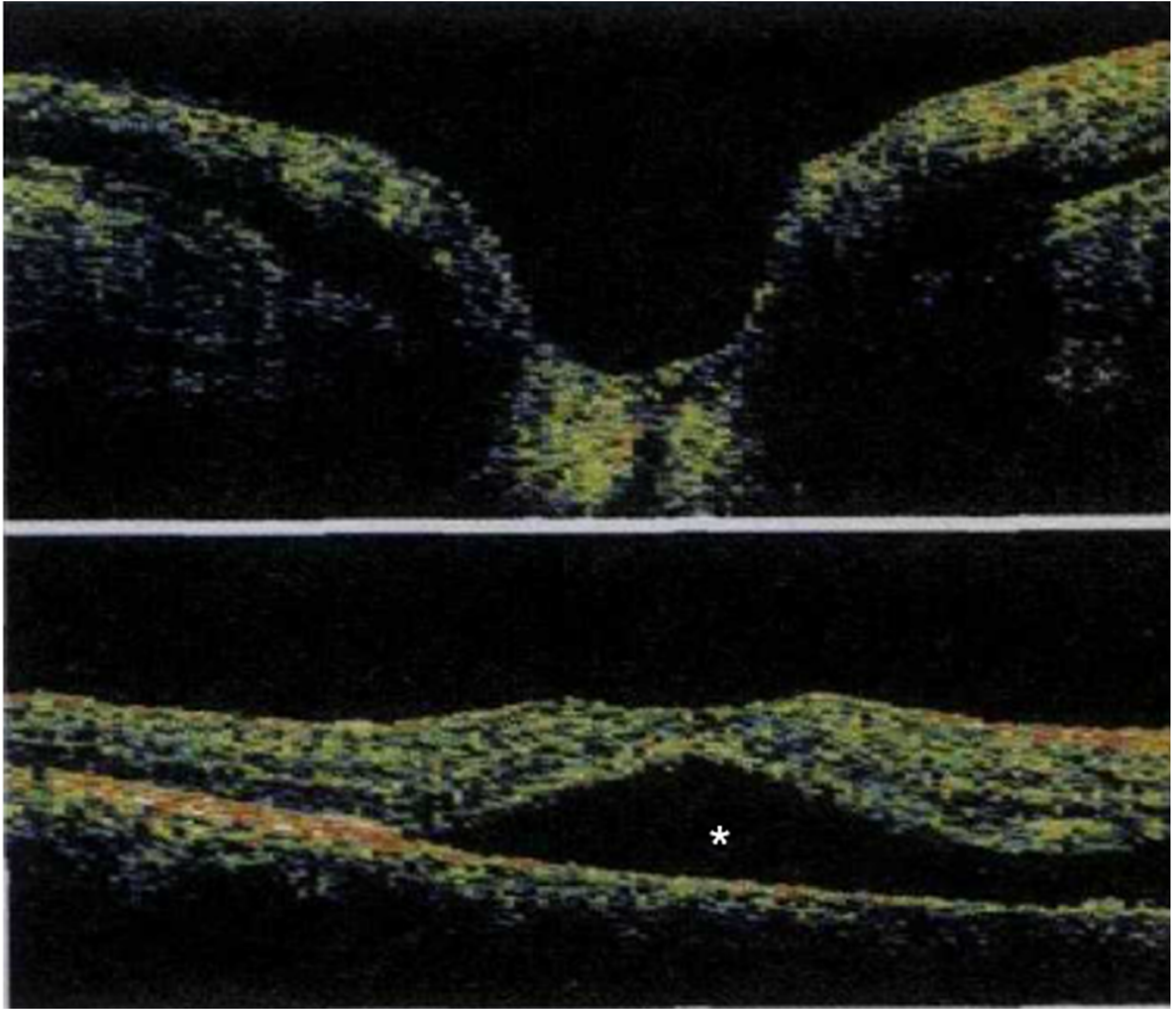
OCT showed serous fluid exudates under the macula (white asterisk)

A 40-year-old woman who first presented with complaints of decreased vision and metamorphopsia in her right eye was diagnosed with CO 9 years ago. At that time her best corrected visual acuity (BCVA) was 12/20. Both eyes had normal anterior segments. Right fundus examination showed a geographic-shaped, yellowish-white choroidal lesion surrounding the optic disc in the right eye (Fig. 1). B-scan ultrasonography of right eye revealed a typical dense echogenic plaque which causing acoustic shadowing behind (Fig. 2). FFA and ICGA had no evidence of CNV except early hyperfluorescent choroidal filling pattern with late diffuse staining. Computerized tomography (CT) showed a hyperdense choroidal plaque with the same density as bone (Fig. 3). Optical coherence tomography (OCT) demonstrated serous retinal detachments at the initial examination (Fig. 4).

There was no evidence of any subretinal neovascularization clinically and the patient maintained stable vision, so she was asked to come for regular follow-up.

**Fig. 5:**
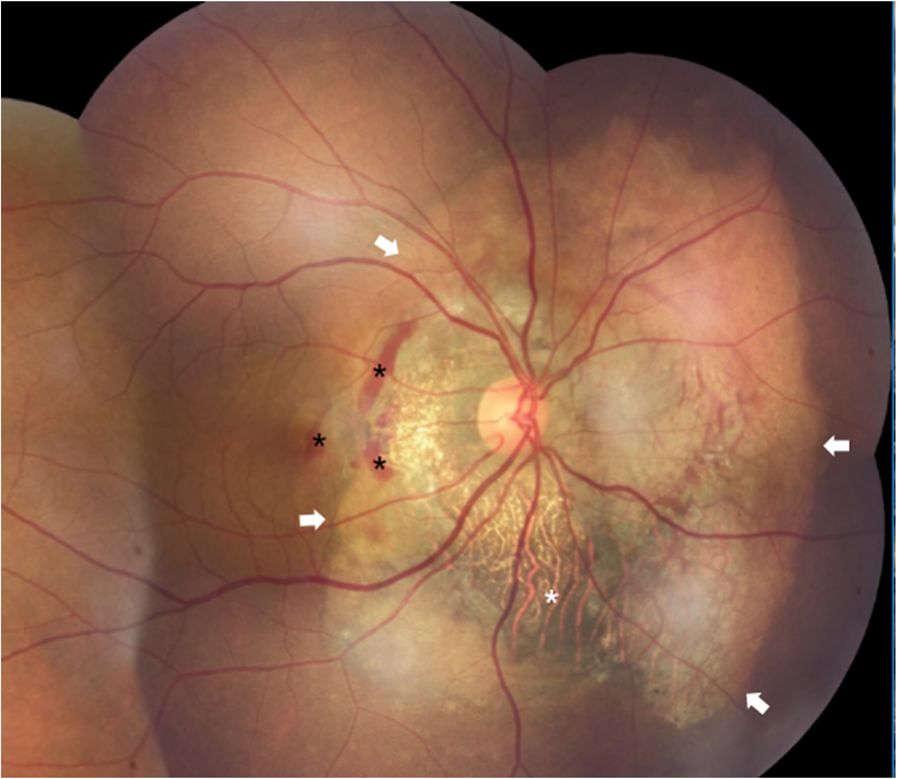
Right fundus showed an enlarged choroidal osteoma in most margins at posterior pole (white arrow) with schistose hemorrhage (black asterisk) beside macula compared to eight years ago. Partial decalcification on the inferior margin and visibility of large choroidal vessels were noted (white asterisk)

**Fig. 6:**
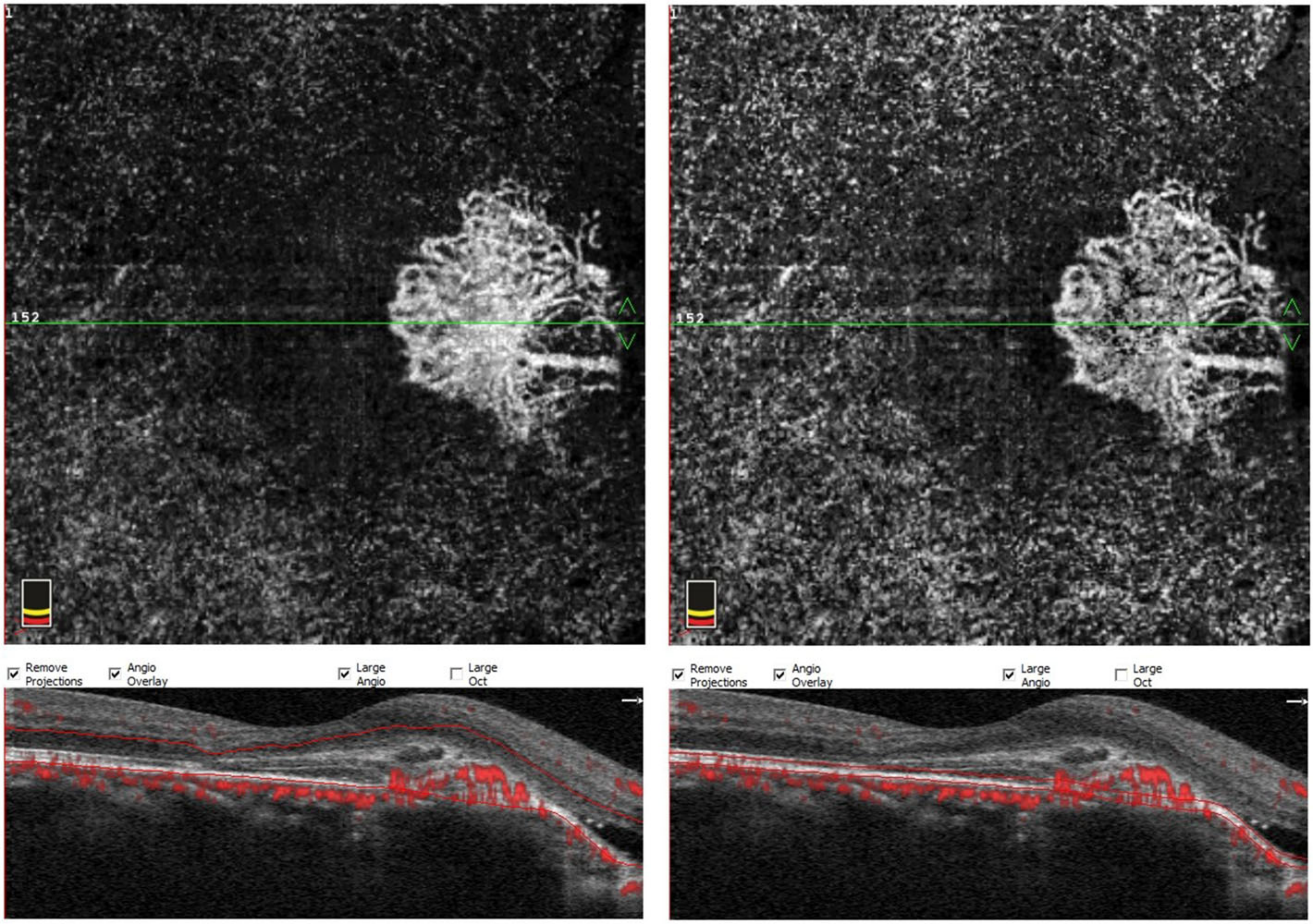
OCTA revealed unique features in the vascular changes of CNV in CO in the outer retinal layer and choroid capillary layers, and subretinal neovascularization (white arrow)

**Fig. 7:**
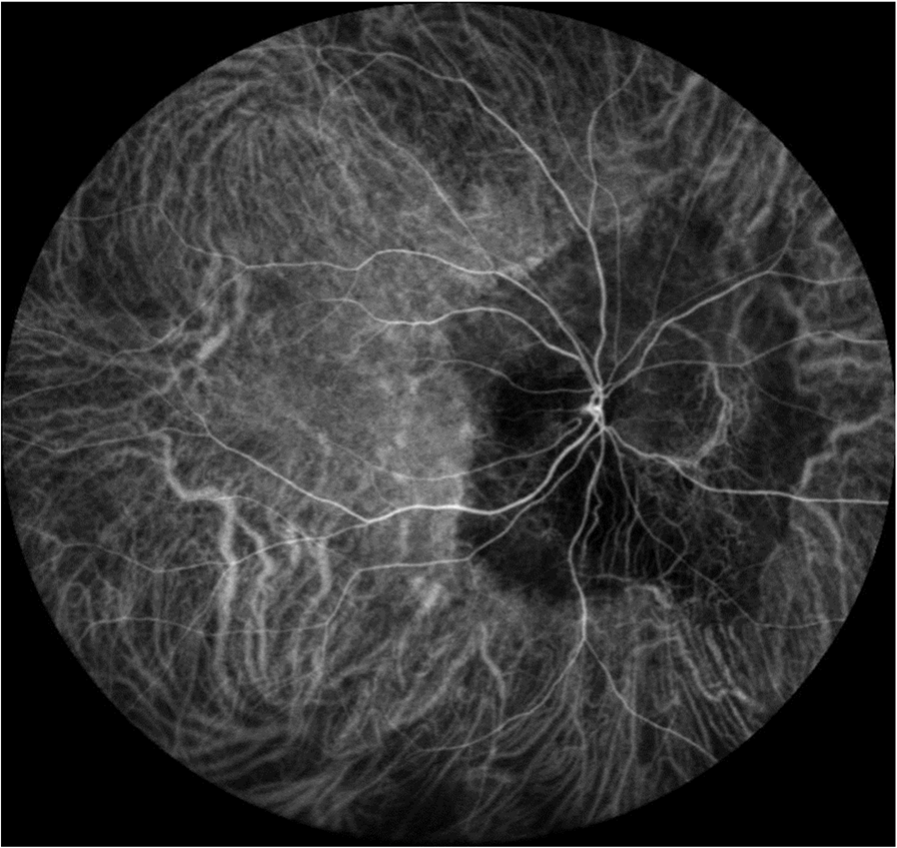
ICGA showed there was hypo-fluorescence at the peripapillary with faint hyper-fluorescence at the macular, corresponding to the location on the fundus photograph. The subretinal neovascularization of CNV was not obvious

Last year, after 8 years follow up, secondary CNV was found by angio-OCT at the temporal of CO. BCVA had decreased to 5/20. B-scan and CT didn’t demonstrate much different than before. Compared to 8 years ago, CO grew up in most margins and macular appeared schistose hemorrhage (Fig. 5). A part of tumor on the inferior margin displayed decalcification and choroid atrophy. OCT revealed subretinal neovascularization with choriocapillaris atrophy. OCTA revealed superficial and deep subretinal neovascularization of CNV in CO not visualize with other imaging methods (Fig. 6). Except CO, ICGA didn’t show the morphology and structure of secondary choroidal neovascularization as obvious as OCTA. (Fig. 7).

**Fig. 8:**
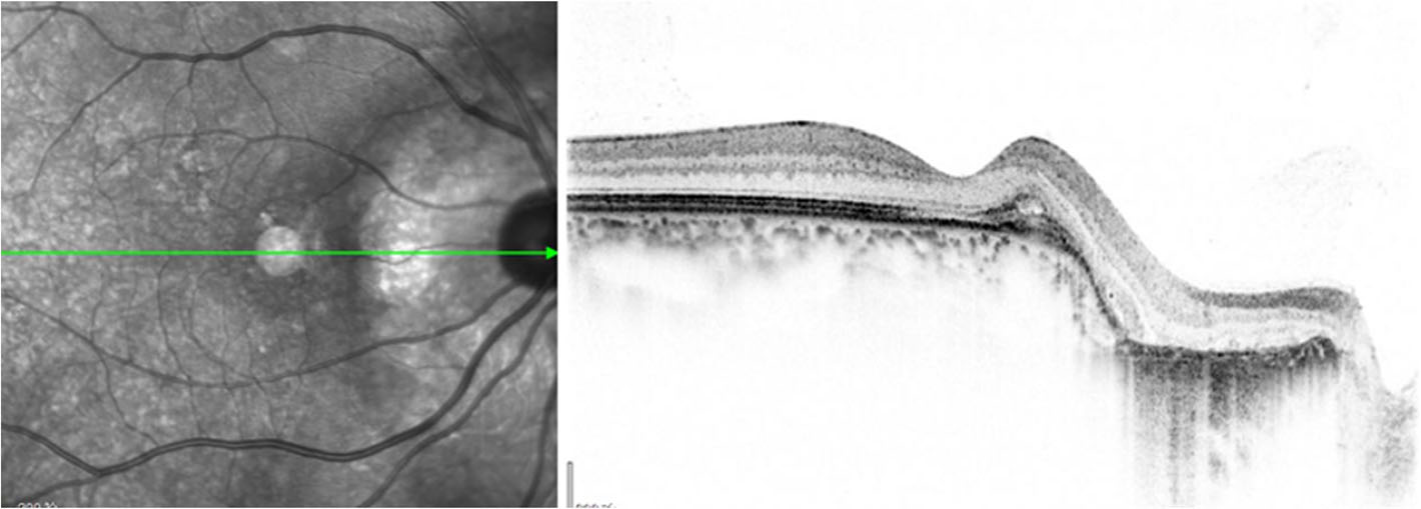
OCT appreciated CNV and on the nasal atrophic retina

**Fig. 9:**
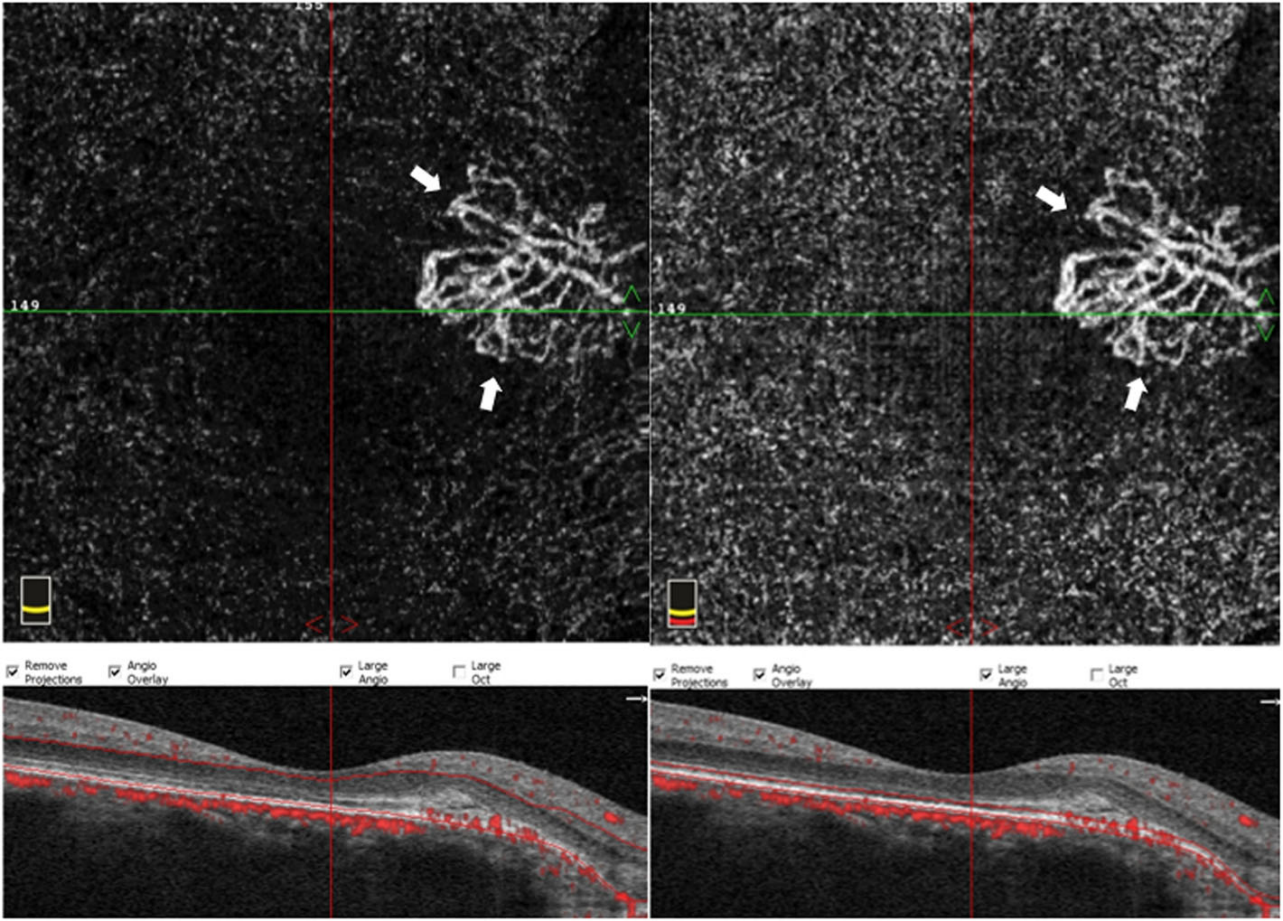
OCTA revealed the regression of choroidal neovascularization density and range in the outer retinal layer and choroid capillary layers. (white arrow)

Based on those findings, the patient was diagnosed with secondary CNV. The patient was administered 3 intravitreal ranibizumab injections at 1-month intervals. In follow-up examination at 3 months post-injections, visual acuity had improved to 18/20 and OCT showed regression of the subretinal fluid (Fig. 8). The patient’s condition was stable during the 1-year follow-up period and no additional injections were required. OCTA also showed reduction in area of CNV (Fig. 9).

## Discussion and conclusion

This case report illustrates the need for prolonged follow-up. The importance of long-term follow-up is even more central to the full assessment and treatment in malignant tumors. Patients with benign tumors also require long-term surveillance to monitor morbidities of intraocular complications. The most important complications of CO are subretinal neovascularization, subretinal and intraretinal hemorrhages, and serous and hemorrhagic retinal detachments. Despite being a benign tumor, the complications may cause serious vision loss, especially CNV, subfoveal fluid, and photoreceptor degeneration [4]. At present, the mechanism by which CNV develops is primarily considered retinal pigment epithelium disruption allows growth of underlying choroidal new vessels together with thinning of Bruch’s membrane and the choriocapillaris, or that osteoma itself has neovascular membrane extensions [5, 6].

Here, we report a case of CO associated with the development of CNV. OCTA showed a dense irregular neovascularization network in the outer retinal layer and choroid capillary layers. Due to serous retinal detachment appearing before CNV, we seem to be considered retinal pigment epithelium disruption tends to be CNV more easily. The presence of subretinal fluid has been found to be predictive of CNV [7]. It is also consistent with the mechanism. A long-term follow-up of choroidal osteoma indicated around 64% of eyes of CO with subretinal fluid resolved spontaneously maintained good vision [8]. Although this is only for those without CNV. In our case, visual acuity improved from 5/20 to 18/20 after 3 monthly injections and was preserved at this level throughout a 12-month follow-up period.

Some reports also describe tumor growth in 41–64% of cases followed for a period of 10 years. Most choroidal osteomas have a slow random growth with an increase in mean basal diameter of around 0.37 mm per year on any of the non-calcified margins [2]. In our case, about 2.96 mm edge should increase compared to 8 years ago according to the report. However, our case didn’t reach the level. On the calcified margins, the growth of CO almost stopped. The cause of the slower growth may be a result of osteoclastic active in the lesion.

There is no standard of treatment for choroidal osteomas in addition to observing, but therapies are directed for complications arising from CNV and subretinal fluid. There are some reports describe the clinical and diagnostic features of this tumor, some of them treat related CNV with photodynamic therapy or/and anti-vascular endothelial growth factor (anti-VEGF) [9, 10]. Some studies showed PDT had achieved success not only in the management of CNV secondary to choroidal osteoma but also preventing tumor growth toward the foveola [10, 11]. However, reperfusion following photodynamic therapy might lead to CNV formation and tumor decalcification [11, 12]. Furthermore, PDT may require more than once treatment and final visual acuity may decline [13]. In addition, thermal laser photocoagulation and transpupillary thermotherapy may have certain efficacy in cases of CNV secondary to choroidal osteoma while increasing retinal damage [5, 14, 15].

Another method of controlling CNV growth is intravitreal anti-VEGF. Our case was treated by anti-VEGF and stable for now, in spite of keeping indispensable monitoring. It is reported bevacizumab and ranibizumab have positive outcomes in both anatomy and visual acuity [9, 16]. The rapid regression of CNV secondary to CO may attributed to enhanced passage of the ranibizumab through the thinned and degenerated RPE and Bruch’s membrane to the subretinal area, thus increasing the drug’s efficacy [16].

This patient did not show any CNV at the first time, after 8 years the tumor got a little bigger and the secondary CNV was founded. We use angio-OCT combine with FFA and ICGA to analysis the characteristics of these changes. On fluorescein angiography, choroidal osteoma has early patchy hyperfluorescent choroidal filling pattern and late diffuse staining due to leakage surrounded by an area of blocked fluorescence. Fluorescein angiography is also helpful in detecting RPE atrophy, CNV formation or the characteristic spider vessels [2, 17]. However, the leaking vessels of the CNV usually are overshadowed by the dye in the late phases [18]. OCTA as a a novel imaging tool which allows the visualization of the retinal and choroidal vasculature, may demonstrate CNV morphology, vessel parameter, the motion of erythrocytes, and even predict treatment response. A small and rarified vascular network inside a capsular formation was observed of CNV secondary to CO [18]. OCTA may reveal some unique characteristics in the vascular changes of CNV in CO.

To conclude, CO is benign ossifying tumors of uveal tract. Most choroidal osteomas have a slow random growth, on any of the non-calcified margins. Lifelong clinical follow-up should be maintained to detect complications at early stages. Therapies directed for complications arising from choroidal neovascularization (CNV) are intravitreal anti-VEGF, PDT and laser photocoagulation. OCTA may visualize some micro vascular lesions instead of other image methods to some extent, and help diagnosis and follow-up of CNV secondary to CO.

## Data Availability

The datasets used and analysed during the current study available from the corresponding author on reasonable request.

## Abbreviations

CO: Choroidal osteoma
CNV: Choroidal neovascularization
BCVA: Best Corrected Visual Acuity
FFA: Fundus fluorescein angiography
ICGA: Indocyanine green fluorescence angiography
OCTA: Optical coherence tomography angiography
CT: Computerized tomography
OCT: Optical coherence tomography
